# Reasons of general practitioners for not prescribing lipid-lowering medication to patients with diabetes: a qualitative study

**DOI:** 10.1186/1471-2296-10-24

**Published:** 2009-04-21

**Authors:** Elisabeth AB, Petra Denig, Ton van Vliet, Janny H Dekker

**Affiliations:** 1 Department of General Practice, University Medical Centre Groningen, University of Groningen, Groningen, The Netherlands; 2 Department of Clinical Pharmacology, University Medical Centre Groningen, University of Groningen, Groningen, The Netherlands; 3 Share Graduate School for Health Research, University of Groningen, Groningen, The Netherlands; 4 Academic General Practice, Groningen, The Netherlands

## Abstract

**Background:**

Lipid-lowering medication remains underused, even in high-risk populations. The objective of this study was to determine factors underlying general practitioners' decisions not to prescribe such drugs to patients with type 2 diabetes.

**Methods:**

A qualitative study with semi-structured interviews using real cases was conducted to explore reasons for not prescribing lipid-lowering medication after a guideline was distributed that recommended the use of statins in most patients with type 2 diabetes. Seven interviews were conducted with general practitioners (GPs) in The Netherlands, and analysed using an analytic inductive approach.

**Results:**

Reasons for not-prescribing could be divided into patient and physician-attributed factors. According to the GPs, some patients do not follow-up on agreed medication and others object to taking lipid-lowering medication, partly for legitimate reasons such as expected or perceived side effects. Furthermore, the GPs themselves perceived reservations for prescribing lipid-lowering medication in patients with short life expectancy, expected compliance problems or near goal lipid levels. GPs sometimes postponed the start of treatment because of other priorities. Finally, barriers were seen in the GPs' practice organisation, and at the primary-secondary care interface.

**Conclusion:**

Some of the barriers mentioned by GPs seem to be valid reasons, showing that guideline non-adherence can be quite rational. On the other hand, treatment quality could improve by addressing issues, such as lack of knowledge or motivation of both the patient and the GP. More structured management in general practice may also lead to better treatment.

## Background

Statins can lower cardiovascular events in patients with diabetes mellitus by around 25%.[1] Treatment guidelines for general practitioners in The Netherlands have recommended prescribing lipid-lowering drugs to almost all patients with type 2 diabetes mellitus. Despite these recommendations, the number of patients being treated has remained rather low. Studies in Dutch general practice showed that many diabetes patients were not receiving statin treatment.[2,3] Low percentages of prescribing are also seen in other countries.[4,5]

Not prescribing medication as recommended by guidelines is sometimes considered suboptimal care but it may be deliberate and justified. Several models have been suggested to explain why physicians do not act according to recommendations. There is the framework as proposed by Cabana et al that recognizes internal barriers, such as lack of knowledge or motivation of the physician, and external barriers, including patient, guideline, and organisational factors.[6] On the other hand, there is the concept of 'clinical inertia' which has been delineated as a problem of the health care professional and the health care system.[7] For quality improvement, it is important to differentiate between clinical inertia and appropriate care.[8] There may be good reasons for not prescribing a lipid-lowering drug in specific cases, such as patient-specific conditions that would preclude the use of such drugs. In addition, it may be the patient who refuses to take treatment, thereby preventing the physician from prescribing the recommended treatment.

Several studies have investigated barriers to prescribing lipid-lowering drugs. [9-11] In general, GPs mentioned concerns about cost, workload, patient compliance and medicalisation as barriers.[9] In patients with ischaemic heart disease, organisational barriers were considered to be important, but also errors and omissions by GPs and patient reluctance or concerns were seen as important barriers.[10,11] It is not known whether the same reasons apply for not prescribing lipid-lowering medication in patients with diabetes.

The purpose of this study was to explore reasons why general practitioners (GPs) do not follow guideline recommendations regarding lipid-lowering treatment in patients with type 2 diabetes mellitus. We used in-depth interviews related to real cases to study the whole spectrum of physician, patient and practice-related barriers as perceived by the GPs.[6,12] Specifically, we tried to gain further insight into the factors that may or may not represent appropriate care, since only the latter should be addressed to improve the quality of care.[8]

## Methods

### Design

We conducted a qualitative study using semi-structured interviews to explore the reasons of general practitioners (GPs) for not prescribing lipid-lowering medication to specific patients with type 2 diabetes mellitus. The research project was checked by the METc office of the University Medical Center Groningen (UMCG) who declared that the study fulfills all requirements for patient anonymity, and was in agreement with regulations for publication of patient data. According to the Dutch Code of Conduct for Biomedical Sciences, all patients with type 2 diabetes mellitus of the participating GPs have been informed that information from their medical records was going to be used for research purposes, and they were given the opportunity to opt out. Less than 0.5% of all patients have been excluded for this reason.

### Participants and setting

Evidence-based treatment guidelines are disseminated among all GPs in the Netherlands. The study was conducted in a region in the north of the Netherlands, where a guideline was distributed in October 2004 that recommended the use of statins in patients with type 2 diabetes. Between October and December 2005, interviews were held with GPs who had indicated that they were familiar with these guideline recommendations. GPs were selected according to a method of purposeful sampling to include variation concerning practice (single-handed or group practice), practice location (city or rural area), and diabetes management (with or without a nurse practitioner). Interviews were conducted until data saturation was reached and no new themes emerged.

### Data collection

Interviews were held in the GP practice by EAB and recorded on CD-rom. All interviews were transcribed verbatim by the interviewer. Before the interview, a coded list of all patients with type 2 diabetes was extracted from the electronic medical records and screened for patients not being prescribed lipid-lowering medication. These coded cases were discussed in the interview, during which the GP was able to access the medical record to identify the coded patient. Starting with an open question, the GPs were asked to elaborate why that particular patient did not use lipid-lowering medication. Possible topics raised included patient-related factors, such as risk factor levels, comorbidity, medication issues, personal circumstances, and GP-related factors, such as beliefs and attitudes towards (preventive) medication, and perceived organisational barriers. The duration of the interviews was approximately one hour. Sometimes not all patients from the list were discussed because of lack of time. At the end of the interview, the GPs were asked if they had any other arguments or reasons for not following the guideline recommendation on lipid-lowering treatment.

### Analysis

The interviews were analysed by EAB and TV using content analysis. Both researchers independently examined the first transcripts before coming together to discuss, modify and agree on a coding frame. After three interviews, a coding frame was developed that distinguished between themes related to the patient as the person deciding not to use medication (patient-attributed factors) and themes that were related to the physician as the one who primarily determined this decision (physician-attributed factors). Within these groups, themes were clustered around different general intentions regarding the use of lipid-lowering drugs. These differentiations were considered relevant for identifying targets and factors that can be addressed in order to improve the quality of care. PD critically examined the framework to improve its clarity and consistency. Transcripts were reread and coded by EAB and TV after the final coding frame was established. Newly collected data were compared with previously collected data and coding. To ensure consistent coding, the two reviewers came together to discuss their final coding. PD reviewed subsets of data to check for accurate coding. Kwalitan version 5.0 was used to support the qualitative analysis.

## Results

In total, three GPs refused to participate for reasons of time constraints. Interviews were conducted with 7 GPs, with no new themes emerging in the last interviews. Of the interviewed GPs, 2 worked in single-handed practices, 4 in a city, and 5 had a nurse practitioner. Three of the GPs were female, and their age ranged from 31 to 55 years.

For each GP, between 10 and 27 patients with type 2 diabetes could be identified that were not being prescribed any lipid-lowering medication, constituting 13–39% of the total number of DM patients in these practices. In total, 16 themes emerged that were grouped in patient and physician-attributed factors. Each GP mentioned between 7 and 10 different themes.

**Figure 1:**
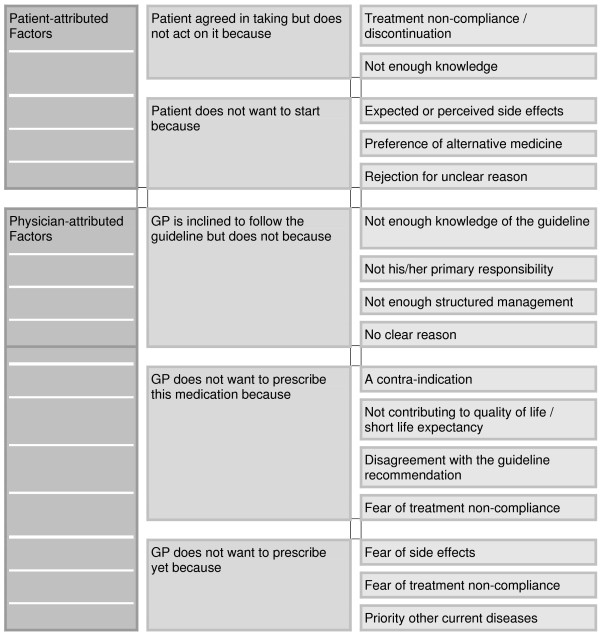
**Patient and physician-attributed factors for not initiating or continuing lipid-lowering therapy**.

Within the group of patient-attributed factors, five themes were grouped in two categories as presented in Figure 1. Most GPs mentioned that although patients initially may agree on taking the medication they do not always act accordingly. This was referred to by some GPs as intentional non-compliance, but also lack of sufficient knowledge or understanding was mentioned as an underlying reason for the patient-attributed discontinuation of medication:

"Oh, I probably said that it [the cholesterol] was alright and then she thought is was alright to stop, something like that, that's possible? That happens: they think everything is in order again." [GP6]

Furthermore, most GPs experienced patients who were not willing to start with this medication at all, because of expected or perceived side effects, preference for alternative medicine or unclear reasons.

"Despite his very serious diabetes, mister Y refused any medication for years. It took me a lot of effort to get him an appointment with our nurse practitioner. He prefers to use earthnuts from Surinam rather than medication." [GP3]

In addition to these patient-attributed factors, there were many concerns and issues raised by the GPs themselves for not being able or wanting to prescribe lipid-lowering medication. Eleven themes grouped in three categories emerged from the analyses (Figure 1). All GPs saw problems of structural or organisational origin which limited the implementation of the guideline recommendation in daily practice. In such cases, the GP was not opposed to prescribing lipid-lowering treatment but had not yet done so for various reasons. These included insufficient knowledge of the guideline recommendation, lack of perceived responsibility, and lack of structured management to initiate timely changes in treatment:

"Yes, mister Z, he just uses eh...yes right, he uses a diet only and he is doing very well. The question is eh, I wouldn't know, should we give people with diabetes, who are doing well on a diet, should they take Zocor [simvastatin]?" [GP2, insufficient knowledge]

"but that patient is monitored by the internist, and I do not regularly check those patients regarding their lipid-lowering medication, because I feel that if they are monitored by the specialist, he has to take care of that." [GP6, lack of perceived responsibility]

"Yes, he was also going to the internist for this. And he has just not started [a statin] then. Also not later. Now he is with our diabetes nurse, and she is using a protocol, so I think he will get one soon." [GP1, lack of structured management]

"Well, if I look at this, I realize I must watch this closer. Because there are a number of people who actually should take a statin" [GP7, lack of structured management].

Furthermore, all GPs questioned the value of lipid-lowering medication in some patients, either because of individual drawbacks or because of general concerns regarding the benefit/risk ratio of this medication in patients with a short life expectancy:

"Considering her prognosis, suffering from breast cancer as well as Alzheimer, this has not even been taken into consideration." [GP4, individual drawbacks]

"Well, people who, eh, have a very short life expectancy, I will not start to bother." [GP5, general reservation relating to short life expectancy]

"So that she might live a month or two longer, at 95? Because that's what we are talking about. Prolonging life, well I think that is nonsense." [GP3, general reservation regarding benefits in the very old]

"So old, and by that I mean at least over 90, but maybe also over 80, ramshackle, and morbid and for that reason have a lower quality of life. Some of these people find it is highly desirable that it ends in a while. That life stops. It is okay. They have lived their life...." [GP2, general reservation related to quality of life]

A more explicit disagreement with the guideline recommendations was also expressed by some GPs, for instance in relation to patients with near goal lipid levels:

"Well, his cholesterol was quite low, it was 4,5...mwah.. then I think, he doesn't really need it, and this is probably not in line with the guideline or standard practice". [GP6]

Furthermore, there were fears of non-compliance mentioned in combination with reserved attitude towards lipid-lowering medication:

"There is a fair chance that she cannot keep track of all her medication and she will make mistakes. I think this doesn't weigh up to the relatively small advantage of a lipid-lowering drug." [GP4]

Fear of side effects or of treatment non-compliance were also mentioned as reasons not to start lipid-lowering medication at that moment:

"...you should not give all at once. Take a new diabetes patient: if you were to follow the guidelines, he would leave your front door with 4 different pills. Patients won't buy that, I think. You have to let them get used to it, little by little. But this gentleman's compliance is very bad, so you have to handle this carefully..." [GP6]

Finally, priorities regarding other current diseases were mentioned by most GPs for not yet wanting to start with lipid-lowering medication:

"Well, it was more a matter of life and death than eh.. worrying about that kind of stuff.." [GP5]

## Discussion

Although the Dutch treatment guidelines recommend prescribing lipid-lowering medication to almost all patients with type 2 diabetes mellitus, around one third of the patients of GPs in our study were not using such medication. This shows that there is a substantial group of high-risk patients that may benefit from lipid-lowering medication but are not receiving such treatment. The reasons for not prescribing lipid-lowering medication to diabetes patients as perceived by GPs could be divided into patient and physician-attributed factors. According to the GPs, some patients just do no follow-up on agreed medication and others object to taking medication in general, partly for legitimate reasons. In addition, the GPs also perceived intrinsic reservations for prescribing lipid-lowering medication in some of their patients, some of which can be seen as appropriate care, and others that are a target for quality improvement. Often, GPs were not opposed to prescribing lipid-lowering treatment but had not yet done so for various reasons.

According to the GPs, some patients influenced their prescribing in a direct way, by not wanting to start or continue using lipid-lowering medication. Patients' reluctance to take medication has been identified before as a barrier for adequate treatment,[10,13] and may be difficult to change. On the other hand, patients' discontinuation of treatment due to lack of knowledge forms an obvious target for improvement. In some cases, patients may have sound reasons for not wanting treatment, such as previously experienced side effects. They may also reject lipid-lowering treatment in general. It has been shown that even well-informed, highly educated patients with diabetes may decide not to take statins.[14] From a shared-decision making point of view, GPs do have to accept such refusals as a legitimate reason for not prescribing. Costs can also preclude patients from taking medication. However, at the time of our study, patients in The Netherlands did not have to pay for this type of chronic medication.

Patient factors also affect prescribing in more indirect ways. GPs expressed a reluctance to start (additional) treatment in patients because of fears of non-compliance. Such concerns have been mentioned before,[9] but it has also been shown that GPs sometimes make inaccurate assumptions and guesses about patients' beliefs and preferences.[13,15] GPs therefore need to check such assumptions with the patient. In some cases, it might indeed be wise not to start too many drugs at once. There can be other priorities in general practice that warrant more immediate attention. Competing demands have been shown to interfere with diabetes care, and general practice has been described as a balancing act that requires prioritization and goal setting by both patient and physician during each encounter.[16] Therefore, postponing the start of lipid-lowering treatment can sometimes be seen as a good decision. From our study results, we can not conclude whether the delay was always reasonable or acceptable. Moreover, little is know about the increased cardiovascular risk created by delay of preventive treatment.

Some patient-related factors form valid reasons for not prescribing a specific drug. As was seen in other populations, medication factors including contra-indications, side-effects and interactions may prevent a physician from prescribing.[8,10] In general, such medication related factors do not preclude GPs from taking appropriate action but in some cases no alternative medication may be available.

Patients' age is a recurrent issue related to undertreatment for cardiovascular diseases.[17] Despite the fact that more evidence is emerging that elderly patients may benefit from statin treatment, many physicians express reservations to prescribing these drugs to patients over 80 years of age. Besides questioning the benefits of lipid-lowering medication in this age group, quality of life and life expectancy clearly play a role in the decision to prescribe preventive treatment.[10] Benefits from treatment are not to be expected in the short term. Patients with a short life expectancy are legitimately not prescribed lipid-lowering medication. All guidelines do recognize this reason for deviating from the recommendations. Prolongation of life is not necessarily wanted, but from our study it is not clear to what extent the patients were actually involved in this decision. Older age in itself should not exclude patients from receiving therapy, but in the frail elderly the risks may outweigh the benefits of starting preventive treatment.[18]

GPs who do not start lipid-lowering treatment in patients with near goal lipid levels, could be viewed as disputing the considerations made in the guidelines. This type of disagreement has been observed before regarding the start or intensification of antihypertensive and glucose-lowering medication in patients with diabetes.[19,20] Reported reasons for inaction in these studies included "patient improving or near goal" and "patient doing well or only borderline hypertension". Also regarding lipid-lowering treatment in patients with diabetes, some GPs expressed that they might postpone prescribing because of marginally elevated risk factor levels.[21] This 'near goal is good enough' attitude was also found to be associated with the decision not to intensify lipid therapy.[22] Although one could defend this attitude, some physicians appear to accept quite high risk factor levels.[19,20] More importantly, for lipid-lowering medication, the cholesterol level should not be the main factor influencing the decision. Statins have shown to reduce cardiovascular risk in diabetes patients regardless of their lipid levels.[1] Our study shows that the doubts that GPs have expressed regarding the value of statin treatment in general,[9] are also present regarding the high risk group of patients with diabetes.

Our study also showed that some GPs are uncertain about the exact recommendations in the guidelines. Although this so-called lack of knowledge could be interpreted as lack of information, it is more likely to be a problem of information overload. Confusing guidelines that are difficult to follow or differ in thresholds for treatment which often change over time have previously been identified as barriers for implementing guideline recommendations.[10] In the Netherlands, this has lead to the development of a national multidisciplinary guideline for cardiovascular risk management that became available in 2006.

Practice organisation is an issue of continuous concern in chronic disease management. GPs see problems within their own practice organisation. Lack of adequate work routines and high workload have been recognized before as barriers to provide adequate coronary prevention.[9,10] When structured management in introduced in general practice, the management of patients with diabetes can improve.[23,24] Furthermore, GPs mentioned problems at the primary-secondary care interface. GP are reluctant to interfere with the treatment when a patient is seeing a specialist. This problem has been observed before as barrier to implement guideline recommendations in general practice.[25]

### Strengths and limitations

This study focussed on the perceptions of GPs, and therefore the role of the patients is only commented upon from their physicians' point of view. We interviewed GPs in one region of the Netherlands who had indicated that they were familiar with the guideline recommendations regarding lipid-lowering treatment in patients with diabetes. Our findings could be limited by this selection but several of the themes that we have captured were quite similar to those observed in other primary care settings. Although the organisation of diabetes care might differ between countries, issues of structured management, shared care, and continuity of care are relevant for most diabetes care settings.[26]

We reached data saturation after 7 interviews, with many themes mentioned by most GPs, suggesting that our purposeful sampling to include variation on practice organization and location was not meaningful. There are no explicit guidelines of determining the saturation point, and it is therefore possible that under representation of specific GPs has affected our outcomes.[27] Although this does not alter the reasons identified in our study, we may have missed some additional reasons for not prescribing lipid-lowering medication to patients with diabetes.

A qualitative approach is seen as the best method for studying reasons underlying treatment decisions. The interviews were held in a non-confrontational way to avoid defensive reactions. We used actual patients to elicit arguments and thoughts of the GPs derived from practice, and not from general professional ideas. This method is relatively new and may encourage more relevant results than a more open interview method.[10]

## Conclusion

Some of the barriers mentioned by GPs for not prescribing lipid-lowering medication to patients with diabetes seem to be valid reasons, showing that guideline non-adherence can be quite rational. On the other hand, the treatment could improve by addressing issues, such as lack of knowledge or motivation of both the patient and the GP. More structured management of these patients in general practice may also lead to better treatment. Finally, this study suggests that the quality of prescribing performance should not be assessed at one point in time, since there may be temporary, valid reasons for not yet prescribing specific medication.

## Competing interests

The authors declare that they have no competing interests.

## Authors' contributions

EAB carried out the interviews, conducted the analyses, and drafted the manuscript. PD participated in the design of the study, assisted in the analysis and interpretation of data, and amended the manuscript. TV conceived of the study, participated in the design, conducted the analyses, and commented on the manuscript. JHD participated in the conception and design of the study, assisted in the interpretation of data, and commented on the manuscript. All authors read and approved the final manuscript.

## Pre-publication history

The pre-publication history for this paper can be accessed here:


